# Enhancing Access to High School Summer STEM Programs Through Proactive Planning and Budgeting

**DOI:** 10.1002/ece3.73069

**Published:** 2026-02-08

**Authors:** Kelsey J. Solomon, Denzell A. Cross, Crystal L. Pendergast, Madison D. McFarland, Krista A. Capps

**Affiliations:** ^1^ Department of Biological Sciences, Institute of Environment Florida International University Miami Florida USA; ^2^ Department of Geosciences Georgia State University Atlanta Georgia USA; ^3^ Odum School of Ecology University of Georgia Athens Georgia USA

**Keywords:** access, budgeting, direct costs, high school summer programs, STEM education

## Abstract

Summer programs are a powerful educational tool for increasing student interest in science, technology, engineering, and mathematics (STEM) careers. However, barriers such as lack of awareness, transportation challenges, and financial constraints can hinder participation. This study examines *Water Dawgs*, a paid summer initiative designed to provide high school students with hands‐on freshwater science education while ensuring accessibility for all interested students. Using *Water Dawgs* as a case study, we explore how proactive planning and budgeting can help mitigate these participation barriers. *Water Dawgs* successfully engaged 16 participants, and survey results indicate increased self‐efficacy in STEM as well as greater awareness of how environmental science impacts daily life and career opportunities. We identify five key barriers—information gaps, resource deficiencies, transportation disparities, food insecurity, and economic limitations—and offer practical recommendations for addressing them through proactive planning and budgeting of direct costs. Strategies include planning and engagement well in advance of the event, allocating direct expenditures to compensate teacher partners and participants for their work, providing all necessary supplies for both classroom and field activities, offering transportation options for all participants, and ensuring access to meals. Our case study highlights the importance of thoughtful program planning and budget development that fully accounts for direct costs associated with removing barriers, making STEM summer programs an option for all interested students.

## Graphical abstract

This study explores how the *Water Dawgs* summer program effectively engaged high school students in freshwater science while addressing common barriers to STEM participation, such as financial and transportation challenges. Through strategic planning and budgeting, the program increased student confidence in STEM and demonstrated how proactive planning can expand access to science education.

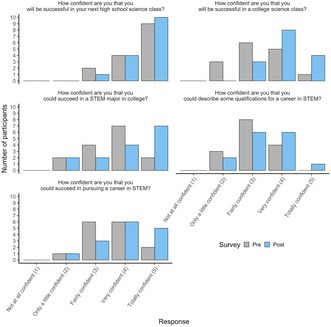

## 1 Introduction

The majority of students who concentrate in science, technology, engineering, and mathematics (STEM) at 4‐year institutions make this decision during high school (Maltese and Tai 2011). College‐ and university‐run high school summer STEM programs are a valuable educational tool to increase the likelihood of pursuing a STEM career (Ashley et al. 2017; Kitchen, Sadler, and Sonnert 2018; Kitchen, Sonnert, and Sadler 2018). Summer STEM programs have been shown to be highly effective in increasing the participation of underrepresented groups in the STEM pipeline and curtailing pipeline attrition (Salto et al. 2014; Rahm and Moore 2016). Even if STEM summer program participants choose not to pursue STEM careers, participation improves STEM self‐efficacy and strengthens overall STEM literacy (Knox et al. 2003; Markowitz 2004). Accordingly, high school summer STEM programs are often developed as a part of the extension or broader impacts components of grants developed by researchers, aiming to provide accessible experiential learning opportunities to all students.

However, barriers can often hinder students from participating in summer STEM programs (Phillips et al. 2007; Brown et al. 2020). For instance, many students may remain unaware of these opportunities due to insufficient marketing or outreach within their communities. Financial constraints are another significant challenge, as high program costs, coupled with the need to prioritize a summer job over unpaid or low‐paid programs, can deter participation (Afterschool Alliance 2021; Lynch et al. 2023). Additionally, logistical obstacles such as limited access to reliable transportation or the absence of provided meals can further restrict access (Phillips et al. 2007; Afterschool Alliance 2021). Even when accepted into these programs, students may encounter cultural and social challenges, such as feelings of isolation or a lack of role models to inspire and support them (LaDue et al. 2024).

Well‐intentioned researchers may not understand some of the barriers facing students from participating in extramural programming. While considerable efforts have been made to address cultural and societal challenges that create barriers to participation in STEM fields (e.g., Ashley et al. 2017; Estrada et al. 2018; Harrison‐Bernard et al. 2020), another critical approach to employ in improving access is to enhance program planning and budgeting. Proactive planning and budgeting can help reduce barriers related to information, finances, and resources, thereby fostering a more supportive and equitable environment in summer STEM programs (Morehead Planetarium and Science Center n.d.). However, there are few resources available to guide program planners in addressing these barriers (but see Urban Libraries Council 2024).

Here, we use *Water Dawgs*, a freshwater science STEM summer program for high school students, as a case study to explore the challenges and solutions associated with planning and budgeting for such initiatives. Specifically, the purpose of this study was to identify five key barriers that can limit student access to participation in high school summer STEM programs and demonstrate how these challenges can be addressed through thoughtful planning and budgeting, drawing on our experiences with *Water Dawgs*. While we anticipated some of these barriers when planning for *Water Dawgs*, others—along with the intensity of their impact—became evident as the program progressed. This case study offers insights into addressing these issues and highlights the importance of proactive budgeting to enhance program accessibility. Through this discussion, we aim to provide actionable recommendations for scientists and others interested in appropriately budgeting the direct costs for a program designed to increase accessibility to summer STEM programs.

## 2 Case Study: *Water Dawgs*

### 2.1 Program Description

*Water Dawgs* was a paid summer program designed to provide high school students, especially those from groups traditionally underrepresented in STEM, with hands‐on education in freshwater science and professional development opportunities. The program was created as part of the broader impacts of a grant that was funded by the National Science Foundation and was designed with three goals. First, it aimed to broaden local participation in summer STEM programs at the university by working with a local public school to recruit students from groups traditionally underrepresented in STEM fields. Second, it aimed to enhance students' understanding of how environmental science issues relate to their everyday lives and future careers, even if those careers fall outside of STEM. Finally, the program sought to boost students' self‐efficacy in STEM disciplines, particularly in environmental science.

To recruit students for *Water Dawgs*, we began efforts 18 months prior to the program's launch in July 2023. We worked with the local school district to identify the schools and teachers who were part of relevant programs who could help connect us with students and families interested in a science summer program. From the subset of interested teachers and their corresponding student populations, we identified a single lead teacher for a nontraditional environmental science program within the public school system that draws students from all high schools in the district. This teacher assisted with student outreach and coordination approximately 8 months prior to the program. Though there were students from all of the public high schools in the district, they were all familiar with one another through their involvement in the county‐wide program. The teacher was financially compensated for their essential contributions to support the program. In collaboration with the lead teacher, we organized three informational events within the school district to advertise the program. These events served to introduce high school students to the instructors and the program, showcase its benefits, and provide an opportunity to distribute program applications. Importantly, the events were designed with parent and caregiver participation in mind, so there was ample time to answer questions and speak with the primary caregivers of the students well before the program start date. At each event, program facilitators shared personal stories about their experiences in science and freshwater research, illustrating the potential impact of pursuing the summer program. To ensure accessibility, recruitment materials were provided in both English and Spanish, reflecting the primary languages spoken by students and their families within the focal school district. After accepting applicants, we distributed surveys to gather information on dietary restrictions and transportation needs.

*Water Dawgs* was held at the University of Georgia (UGA) in Athens, Georgia, USA, during a 10‐day period in the summer of 2023 with 16 student participants. We developed the *Water Dawgs* curriculum, which consists of twenty 3.5‐h learning modules, and allows for the program to be designed to the needs of the participants and instructors. It can be implemented in a variety of ways, depending on the nature of the program (e.g., fully as either a 2‐week program of full days, a 4‐week program of half days, or individual units). For the summer program, we conducted a 10‐day, full‐time program that ran from 8:30 a.m. to 4:30 p.m.

All learning modules are based on the Biological Sciences Curriculum Study (BSCS) 5E instructional model (Bybee et al. 2006) and cover a variety of topics across freshwater science, water quality monitoring, professional development, and college readiness. Programming included field trips to a local stream for hands‐on educational activities. All *Water Dawgs* instructional materials, as well as instructor support resources (including the instructor guide, materials lists with costs, surveys, and parent meeting information) are freely available online in both English and Spanish (Solomon and Capps 2025a; translation, Veronica Choque Campos and Krista Capps).

Before going into the field with participants, the program activities and all instructors were vetted and approved through the Programs and Activities Serving Minors background check and training program at the university. Additionally, the lead primary investigator (PI) held a comprehensive meeting to review key logistical, safety, and accessibility considerations. We collected information from each student about their physical abilities, including allergies, driving, swimming, and walking capacities, and made plans accordingly. The week before the program, we considered the weather forecast and changed the timing of some of the activities so they would occur during cooler parts of the day.

While none of the students were physically, vision, or hearing impaired, we had developed plans to accommodate these needs prior to the program. Sites were selected to ensure participation for students who could not swim, and all students were supplied with appropriate field gear and necessary equipment. We also outlined meal plans, water needs, and restroom access for field and laboratory activities for the students at the beginning of each day of the program. Potential environmental hazards and social risks were identified and discussed with the group, and students were advised on suitable clothing for the expected weather. We provided space for participants to express any concerns, including confidential conversations with supervisors.

Prior to the program, emergency contact information for program staff and primary caregivers was exchanged, a group was set up through a social networking messaging app for announcements to students, and check‐in procedures were established, with regular updates arranged through designated contacts. Program staff set up a transportation schedule that was shared with the parents, as it began 1.5 h before the start and after the end of the program each day to account for travel times and traffic.

**FIGURE 1:**
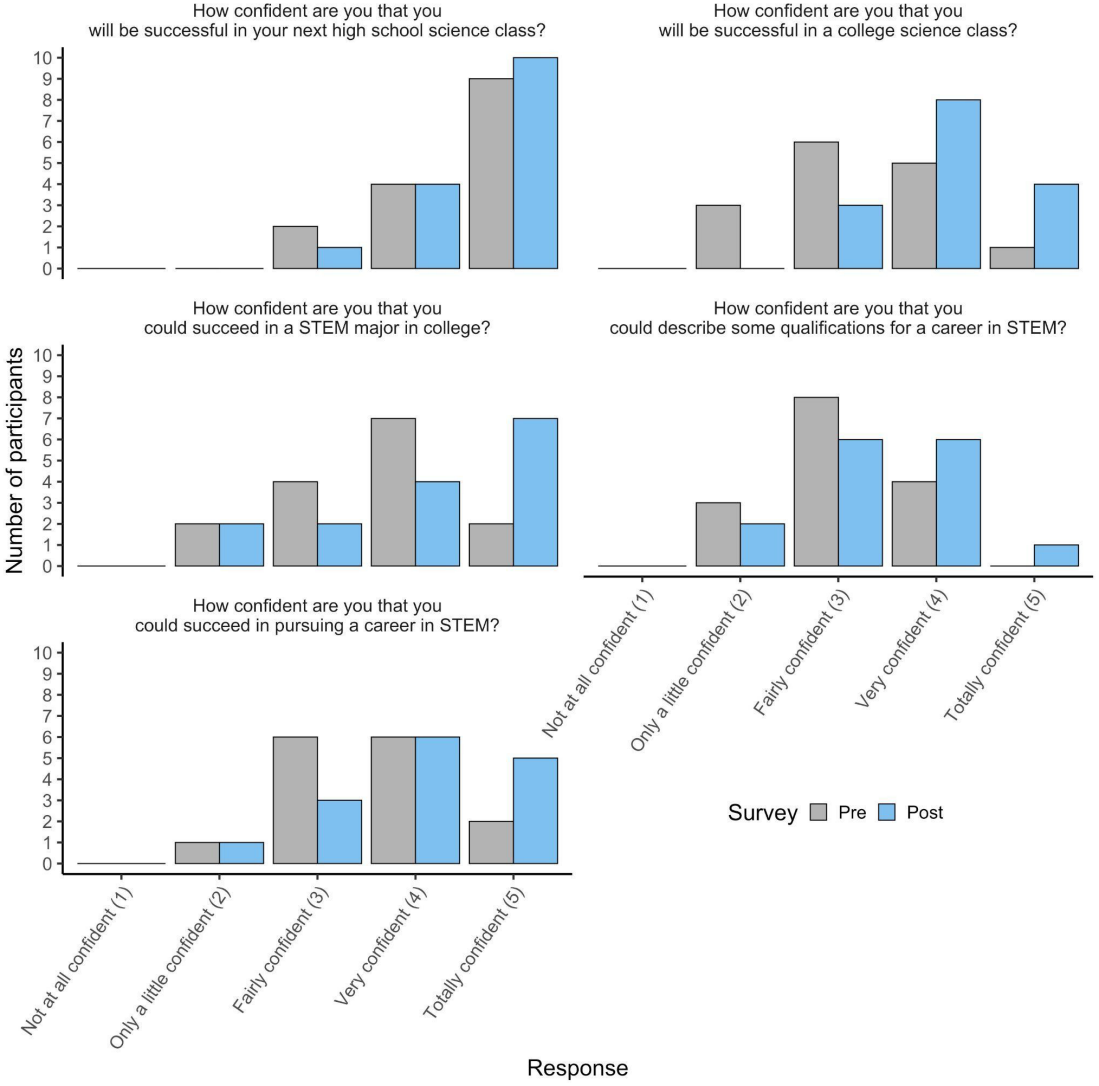
Student responses (*N* = 15) to survey questions assessing STEM self‐efficacy before and after the program. Self‐efficacy was measured using confidence ratings on a Likert scale ranging from 1 (not at all confident) to 5 (totally confident).

To evaluate program outcomes, students completed two surveys. The first survey measured STEM self‐efficacy using a 5‐point Likert scale, where 1 indicated “not at all confident” and 5 indicated “totally confident.” This survey was administered at both the beginning and end of the program to assess the effect of the program on STEM attitudes. We calculated mean scores and standard deviations for each question to analyze these changes, rather than track individual responses in order to maintain anonymity (Figure 1). Because survey responses were analyzed in aggregate and no individual‐level changes were tracked, the project was Institutional Review Board (IRB) exempt. One student, who only completed the end‐of‐program survey, was excluded from the analysis to maintain consistency. A second survey, administered anonymously at the conclusion of the program, included questions related to financial considerations and realities associated with participation to evaluate challenges and barriers to participation (Table 1). It also explored students' career aspirations in ecology and environmental science, as well as their interest in post‐program internship opportunities (Table 2). At the end of the program, students received $1000 to compensate them for their full‐time participation in the 10‐day program.

**TABLE 1:** Student responses (*N* = 16) to survey questions regarding motivation, stipend, and employment during the program. Students were prompted to check all responses that applied.

Question	Response	Number of responses
1. Motivation and stipend: Please check all that apply	The stipend was the primary motivation for me to participate in the program	1
	I was interested in the stipend and I was interested in the content of the program (environmental science)	14
	I was primarily interested in participating for the experience and learning about environmental science	6
	I could not have participated in the program without the stipend because I have to be earning money during this time	2
	I could have participated in the program without receiving the stipend	4
	I was interested in the program because it might allow me to receive a letter of recommendation for college	7
2. Employment during the program: Please check all that apply	I worked a part‐time job during the *Water Dawgs* program (< 40 h per week)	4
	I worked a full‐time job during the *Water Dawgs* program (~40 h per week)	3
	I did not work a job during the *Water Dawgs* program	9

**TABLE 2:** Student responses (*N* = 16) to survey questions regarding study/career aspirations in ecology/environmental science and a post‐program internship opportunity.

Question	Response	Number of responses
1. Considering studies/a career in ecology or environmental science. Please check all that apply	I **was** considering studies or a career in ecology or environmental science before *Water Dawgs* and **I am still interested** in pursuing studies or a career in ecology or environmental science.	5
	I **was not** considering studies or a career in ecology or environmental science before *Water Dawgs* and **I am still not interested** in pursuing studies or a career in ecology or environmental science, **but I can now understand** how learning concepts in ecology and environmental science could apply to my intended career.	11
	I **was not** considering studies or a career in ecology or environmental science before *Water Dawgs* and **I am still not interested** in pursuing studies or a career in ecology or environmental science.	0
	I **was** considering studies or a career in ecology or environmental science before *Water Dawgs* **but I am no longer interested** in pursuing studies or a career in ecology or environmental science.	0
2. If a paid internship position with flexible after school hours was available during the 2023–2024 school year to work in the Capps Lab (Dr. Krista's Lab) in environmental science, I would be interested in this position	Yes	13
	No	0
	Maybe	3

*Note:* For Question 1, students were prompted to check all responses that applied. Bold underlining was used in the survey to ensure that students noticed and understood the key differences among response options.

### 2.2 Participant Selection, Demographics, and Motivations

We were financially and logistically prepared to support approximately 20 students in the program and were hoping to maintain a student‐instructor ratio of 5:1 or 4:1. Due to our targeted recruitment, a total of 21 students applied to the program, and all were accepted. However, some applicants were not able to participate in the full program, and the final *Water Dawgs* class included 16 participants from the Athens area, all between the ages of 15 and 17, who anonymously self‐categorized into a variety of demographic fields, many of which have been classified as underrepresented in STEM fields by the federal government (National Center for Science and Engineering Statistics 2019). All students were interested in attending a college or university.

Financial considerations and realities were paramount in the decision of the students to apply for the program, the ability of the students to participate for the duration of the program, and in influencing the proportion of students working full or part‐time jobs during the STEM program. When asked to detail their primary motivation for participation and evaluate the influence of financial recompense as a primary motivator, seven respondents stated that they were primarily motivated by the opportunity to gain experience and learn more about environmental science, while only one student indicated that the stipend was their main reason for participating (Table 1). A great majority of the students, 14 out of 16, indicated that they were drawn both by the stipend and their interest in the program's content (environmental science; Table 1). Additionally, students also indicated that they were interested in participating to support their application process to colleges. In their responses, seven students noted that they were interested in the program because it could potentially lead to a letter of recommendation for college (Table 1). When asked specifically if the stipend was required for a student to participate in the program, two of the 16 students indicated that they would have been unable to participate, as they needed income over the 10‐day period, while only four students explicitly stated they could have participated in the program without the stipend (Table 1). Notably, almost half of the participants (7 of 16) held full or part‐time jobs while participating in our eight‐hour‐a‐day training program. Three students held full‐time jobs (approximately 40 h per week), four students worked part‐time jobs (fewer than 20 h per week), and nine students reported that they did not have work scheduled during the 10‐day program (Table 1).

### 2.3 Program Impact on Career Interests and STEM Self‐Efficacy

At the conclusion of *Water Dawgs*, all participants indicated the program had either reinforced their interest in pursuing careers in ecology or environmental science or that the program had made them aware of how their own career interests were tied to environmental conditions (Table 2). The five students who expressed interest in pursuing studies or a career in ecology or environmental science prior to the program remained interested in pursuing these paths after participating (Table 2). Although we did not collect specific information about students' future career goals, a subset of the 11 students who reported no interest in studying or pursuing careers in ecology or environmental science both before and after the program provided contextual comments in response to other questions indicating interest in a range of different fields, including but not limited to psychology, social work, public health, medicine, and law. These students now reported a better understanding of how concepts from ecology and environmental science could be relevant to their intended career paths (Table 2). When asked about participating in a future paid environmental science internship with flexible after‐school hours in a program leader's laboratory, 13 students expressed interest, and three students said they might be interested (Table 2).

Students reported an increase in self‐efficacy in STEM throughout the program (Figure 1). When asked about their confidence in succeeding in a college science class, the average (±SD) confidence score rose from 3.3 ± 0.9 at the start of the program to 4.1 ± 0.7 by the end. Regarding their confidence in succeeding in a college STEM major, the score increased from 3.6 ± 0.9 to 4.1 ± 1.1. Students also showed greater confidence in pursuing STEM careers. When asked if they could confidently describe the qualifications needed for a STEM career, their response increased from 3.1 ± 0.7 to 3.4 ± 0.8. Additionally, responses about confidence in pursuing a STEM career rose from 3.6 ± 0.8 to 4.0 ± 0.9. Confidence in succeeding in their next high school STEM class was high at both the beginning (mean: 4.5 ± 0.7) and end (mean: 4.6 ± 0.6) of the course. This was unsurprising as we had, with support of the teacher, intentionally recruited students who had previously demonstrated an interest or success in STEM courses.

## 3 Addressing Barriers to Accessing Summer STEM Programs: Planning and Budgeting Solutions

Through our experience with *Water Dawgs*, we identified five major barriers that could limit access to student participation in high school summer STEM programs that can be addressed with appropriate budgeting of direct costs (Table 3).

**TABLE 3:** Example direct‐cost budget for a 2‐week, full‐time summer STEM program involving 10 participants, one undergraduate assistant, two graduate assistants, a community liaison, and translation services.

Budget category	Budget item	Cost per unit	Example budget
Stipends	Program participants	$100 per day per participant	$10,000
	High school teacher partner/community liaison	$35 per hour	$1000
	Undergraduate assistant	$17 per hour	$2400
	Graduate assistant(s)	1 month of summer salary per assistant	$12,000
Rentals	Facilities (including computer use)	Rental cost through university	$1000
	Rental van	$65 per day plus gasoline	$2000
Supplies	Program supplies (not including laboratory equipment)	$600 per participant	$6000
	Field apparel and supplies	$75 per participant	$750
	Food (lunches and snacks for 10 days)	$15 per day per participant	$1500
Other	Translation services for program materials	$20 per hour	$1000

*Note:* The table includes per‐unit costs and a sample budget based on these program parameters. Costs are provided as illustrative examples and will vary based on the number of student participants, undergraduate and graduate pay rates, rental fees, and other local factors. See Solomon and Capps (2025a) for further breakdown of program supply estimates.

### 3.1 Addressing Information Gaps: Overcoming Barriers to Awareness in Summer STEM Programs

To overcome barriers to awareness about summer STEM training and work opportunities, we recommend allocating a portion of direct costs to compensate teachers or program directors for work assisting with program promotion, student recruitment, and communication with families (Table 3). For *Water Dawgs*, the teacher we partnered with played multiple key roles in program success. First, they were instrumental in advertising and recruitment, leveraging their familiarity with students to identify those who were hoping to pursue a college degree and were either interested in pursuing a career in environmental science or were performing well in STEM courses. Second, they promoted the program to both the students and their families. Third, they served as a trusted intermediary between *Water Dawgs* facilitators and the students and their families. By maintaining ongoing communication during the recruitment process, the teacher enabled multiple touchpoints with students—answering questions and providing support beyond a single recruitment event—which strengthened our recruitment efforts for the program. Teachers or program directors should be compensated for this work (Table 3), as these efforts extend well beyond the 40‐h work week and their traditional teaching responsibilities. Compensation also supports teacher investment in the program's success. We estimate that our lead teacher provided at least 40 h of support for the program prior to the program's inception.

To make recruitment events effective for everyone, we recommend taking additional steps to increase accessibility and engagement. For example, the lead PI was bilingual and conducted family meetings in English and Spanish, the two primary languages spoken by our target participant community. If researchers are advertising to audiences that primarily speak another language, they should consider budgeting for translators to ensure recruitment and advertising materials are available in the primary language(s) spoken by the students and their families (Table 3).

In advertising the program, it is also important to highlight the broader impacts of your program for your participants. *Why is it valuable even if students pursue a career in medicine or a career outside of STEM fields?* Emphasize the long‐term benefits of the program such as critical thinking skills, increased confidence in content knowledge, STEM awareness, and improved problem‐solving abilities in your recruitment materials. When possible, encourage participation from a group of program instructors that have similar demographic backgrounds to your target group of participants (Egalite and Kisida 2017; Arif et al. 2021). In the case of *Water Dawgs*, we found it helpful for all program leaders to share personal stories and their diverse experiences while also explaining the larger significance of freshwater science to their well‐being and to their communities. These additional efforts seemed to engage students who might not have otherwise considered participating in the program.

### 3.2 Addressing Resource Deficiencies: Overcoming Barriers due to Lack of Gear and Supplies

To ensure all students can participate safely and comfortably, we recommend carefully budgeting for and providing the necessary supplies and resources to complete your training (Table 3). This helps eliminate resource barriers among participants and allows students to fully engage in the program. For example, because *Water Dawgs* includes both classroom and field components in aquatic environments, we ensured students had the appropriate materials for both settings. In the classroom, we budgeted for basic school supplies like pencils, notebooks, and binders, and we made sure to have access to computers to support instruction. Access to computers was included in our budget as indirect costs. However, researchers are able to request funding for access to computing resources through rental agreements as part of a direct cost request.

For field work, we supplied materials such as rubber boots and sampling equipment, and items for outdoor safety, such as water bottles, field safety gear, bug repellent, and sunscreen. While not all STEM programs involve fieldwork, they may have other specialized components, such as lab activities, that require specific resources, and these should be included in your budget. From a practical standpoint, providing required supplies and requiring participants to leave them in our space at the university also ensured that students did not forget their own supplies at home, preventing them from participating. To reduce program and supply costs, organizers may seek donations from companies, universities, or laboratory facilities when possible and reuse durable supplies across multiple program years.

Beyond physical supplies, we also recommend critically considering the facility and staffing needs during budget development. For example, *Water Dawgs* was held on the University of Georgia's campus, where we had to secure a dedicated space and access to restrooms that were restricted from access by other adults who had not completed the required training and background checks needed for working with minors. Again, these costs were budgeted as part of our indirect costs. However, if needed, rental fees associated with access to appropriate space can be included as direct costs in the budget (Table 3).

Staffing considerations are also crucial. Though some university spaces are free to use and are accounted for in indirect cost return and student volunteers and outreach programs may provide instructor support, ensuring programs have an appropriate space and enough vetted staff to support participant needs is essential and should be accounted for in program budgets (Table 3). In our case, we had participants with varying physical abilities, which influenced their abilities to participate in field work. This required us to have an additional program facilitator in the field to ensure all students could fully and safely engage in the program. Taking facility and staffing needs into account during planning will help program leaders account for direct costs for staff that need to be included in the budget.

### 3.3 Addressing Transportation Disparities: Overcoming Barriers due to Lack Reliable Transportation

To ensure equitable access to transportation, it is important to plan alternatives to parent/guardian drop‐offs and pickups (Urban Libraries Council 2024). We recommend budgeting for vehicle or van rentals as travel costs or providing participant stipends for public transportation based on the program and student needs (Table 3). If public transportation is readily available in the area and caregivers and students feel comfortable using this resource, mapping routes for participants can also be helpful. If public transit is limited or participants are too young to travel on public transportation alone, renting a vehicle and organizing a driver may be necessary. In such cases, there are additional university, local, and state regulations regarding the transportation of minors that need to be accounted for in the budgeting process. For instance, these regulations can impact the type and size of vehicle rented or the number of participants allowed in the vehicle. This may impact the overall rental costs and the costs associated with providing drivers who have been legally vetted. For *Water Dawgs*, the public transportation schedule was not a viable option to support the timing of our program activities, making vehicle rental and transport of the participants the only feasible solution. We did not allocate enough funding to pay a driver; therefore, the PI transported all of the participants who lacked transportation to and from the program each day, which totaled approximately 30 h of driving during the 10‐day training period.

Clearly communicating transportation options during recruitment and gathering participant needs during onboarding can help with planning, but it is also important to remain flexible as transportation needs may change leading up to and throughout the program. For instance, during *Water Dawgs* onboarding the month before the program, only two of the 16 participants initially required transportation. However, a week before the program began, five additional students requested transportation assistance. As a result, we had to secure a larger van, begin pick up times earlier, and extend drop off times later by 1 h. We also had to change all of our transportation communication to the participants and their caregivers right before the program to accommodate all of the students. If researchers are relying on drivers within their own team, it is essential that most, if not all, instructors are permitted by the university to transport minors in university and nonuniversity vehicles.

Transportation logistics may also change throughout the program. Throughout our program, students who were working part or full‐time jobs often needed to change their pickup or drop‐off locations. This impacted the order and timing of the transportation schedule on a daily basis. We recommend that program coordinators specifically discuss transportation needs and logistics with families during the initial planning stage. Explain some of these challenges and emphasize how the challenge is magnified with each request so that families are aware of the complexity of the issue and plan accordingly prior to the onset of the program. By anticipating these factors and maintaining flexibility, programs can better support participants and program leaders and ensure transportation is not a barrier to access and that transportation needs do not exceed the capacity of the leadership team.

### 3.4 Addressing Food Insecurity: Overcoming Barriers due to Lack of Access to Meals

To ensure equitable access for food, we recommend budgeting for meals and snacks for all program participants, especially if the participants are students participating from districts where a large proportion of the population is eligible for free breakfast or lunch programs (Table 3). The rate of poverty in Athens‐Clarke County is high, with 26.3% of the population living below the poverty line in 2023 (U.S. Census Bureau 2024), and our public school system provides free breakfast and lunch to all students (Clarke County School District n.d.). *Water Dawgs* was an all‐day program targeting participants from Athens; accordingly, we provided early morning and afternoon snacks and lunch to the students daily as part of the direct participant support costs in our budget. There are a range of options to provide meals. In our case, we supplied food from grocery stores and students made their own lunches. This activity had to be built into the program schedule to account for lunch preparation time. Alternatively, programs could provide meals by catering them or, if the program is hosted at a college or university campus, costs could be budgeted to allow participants to have vouchers for dining hall access. Keep in mind that the food the program provides may be the students' primary meal(s) of the day, so we recommend planning the quality and quantity of food provided accordingly. If the programming team needs more information to create a realistic food budget, a conversation with the supporting teacher may provide additional insights to issues with food insecurity for the students involved in the program. To reduce program costs while still ensuring an adequate quantity of food for participants, organizers may consider requesting food donations from local grocery stores or caterers, or seeking voucher support from universities for use in campus dining facilities.

### 3.5 Addressing Economic Limitations: Overcoming Barriers due to Students' Need to Earn Income in the Summer

To encourage participation from students who might otherwise have to choose between a summer job and a STEM program, we recommend offering financial compensation to participants for the work they are doing in the program (Table 3). For *Water Dawgs*, we compensated participants for their work with a $1000 stipend for the 10‐day program. A written agreement that was signed by the participants and their legal guardians before the program specifically stated that any participant missing more than 2 h of the 80‐h program would not receive any compensation. We explained this policy again to participants on the first day of the program, and participants were required to recommit to this agreement by reading and signing it again on the first day of the program. We subsequently sent an email reminding their families and the teacher about this policy.

For our program to work safely with program staff, we could not support students arriving late or leaving early to campus, and we had designed activities to support specific group sizes. Therefore, our compensation agreement was all ($1000) or nothing ($0). This agreement was presented and the justification was explained at all in‐person planning meetings with students and their families before the program, and in multiple emails to all potential participants. In response, we had four participants withdraw from the program before the program began, and one person attended the first day and withdrew from the program. The remaining 16 students participated in the entire program. Although we did not collect additional information from the students who withdrew from the program or their families, requiring students to sign contracts prior to program initiation may have reduced the overall number of withdrawals. Nevertheless, the final number of participants remained within our target student‐to‐instructor ratio (i.e., 4:1). Additionally, we were logistically unable to accommodate new transportation requests after the program start date, an issue that we know affected at least two students who withdrew approximately 48 h before the program began.

Financial compensation for program participation was crucial in recruiting students from our target public school program. Among the 16 participants, two indicated that they would not have been able to participate without the remuneration (Table 1). Additionally, compensation for work served as a key motivator—one participant cited it as their primary reason for joining, while 14 others expressed interest in both the compensation and the program's content (Table 1).

To maximize the impact of compensation, we recommend clearly outlining the details in recruitment and onboarding materials. These should include eligibility criteria, the total compensation available to each participant, the payment schedule, and the payment method. It is also important to explain the rationale behind offering compensation. In our case, participants were expected to actively engage in the program and put effort into learning the content. Accordingly, they received compensation for their work. Transparent and consistent verbal and written communication with teachers, students, and their families ensures that prospective participants understand both the expectations for participation and the financial support available. This clarity allows them to make informed decisions about their involvement.

## 4 Conclusion

This study demonstrates how high school summer STEM programs like *Water Dawgs* can effectively increase environmental literacy while being designed to account for costs that remove barriers to participation. Survey results from *Water Dawgs* showed that 100% of participants recognized the value of learning about ecology and environmental science—either due to an interest in pursuing a career in the field or because they saw its relevance to their potential career paths (Table 2). The *Water Dawgs* summer program successfully achieved its goals by engaging students from a target population in a local public school and removing barriers that would have prevented many from participating in a summer STEM training program. Participants also gained a deeper understanding of how environmental issues connect to their daily lives and future careers (Table 2) and reported increased STEM self‐efficacy by the end of the program (Figure 1). This study highlights how many barriers to participation in high school summer STEM programs can be mitigated through careful planning and budgeting. It highlights the importance of accounting for direct costs that are often overlooked when designing activities intended to achieve broader societal impacts associated with scientific research.

## Author Contributions

**Kelsey J. Solomon:** data curation (equal), formal analysis (lead), validation (lead), visualization (lead), writing – original draft (lead). **Denzell A. Cross:** data curation (supporting), investigation (equal), project administration (supporting), writing – review and editing (supporting). **Crystal L. Pendergast:** investigation (supporting), writing – review and editing (supporting). **Madison D. McFarland:** investigation (supporting), writing – review and editing (supporting). **Krista A. Capps:** conceptualization (lead), data curation (equal), funding acquisition (lead), investigation (equal), methodology (lead), project administration (lead), supervision (lead), writing – review and editing (equal).

## Conflicts of Interest

The authors declare no conflicts of interest.

## Data Availability

The data and code supporting the findings of this study are available in the Environmental Data Initiative (EDI) Data Portal (Solomon and Capps 2025b) [https://doi.org/10.6073/pasta/a0ac536a973420f2e27bc73cfa0d505e]. The *Water Dawgs* curriculum can be accessed here: https://github.com/kjsolomon/water‐dawgs (Solomon and Capps 2025a).
